# Protist taxonomic and functional diversity in aquatic ecosystems of the Brazilian Atlantic Forest

**DOI:** 10.7717/peerj.15762

**Published:** 2023-08-01

**Authors:** Vanessa Carvalho da Silva, Noemi Fernandes

**Affiliations:** Instituto de Recursos Naturais, Programa de Pós-Graduação em Meio Ambiente e Recursos Hídricos, Universidade Federal de Itajubá, Itajubá, Minas Gerais, Brazil

**Keywords:** Coastal lagoons, DNA metabarcoding, Eukaryotic diversity, Inland ecosystems, Neotropics, South America, Protist diversity

## Abstract

The Brazilian Atlantic Forest and its associated ecosystems are highly biodiverse but still understudied, especially with respect to eukaryotic microbes. Protists represent the largest proportion of eukaryotic diversity and play important roles in nutrient cycling and maintenance of the ecosystems in which they occur. However, much of protist diversity remains unknown, particularly in the Neotropics. Understanding the taxonomic and functional diversity of these organisms is urgently needed, not only to fill this gap in our knowledge, but also to enable the development of public policies for biological conservation. This is the first study to investigate the taxonomic and trophic diversity of the major protist groups in freshwater systems and brackish coastal lagoons located in fragments of the Brazilian Atlantic Forest by DNA metabarcoding, using high-throughput sequencing of the gene coding for the V4 region of the 18S rRNA gene. We compared α and β diversity for all protist communities and assessed the relative abundance of phototrophic, consumer, and parasitic taxa. We found that the protist communities of coastal lagoons are as diverse as the freshwater systems studied in terms of α diversity, although differed significantly in terms of taxonomic composition. Our results still showed a notable functional homogeneity between the trophic groups in freshwater environments. Beta diversity was higher among freshwater samples, suggesting a greater level of heterogeneity within this group of samples concerning the composition and abundance of OTUs.Ciliophora was the most represented group in freshwater, while Diatomea dominated diversity in coastal lagoons.

## Introduction

It is widely known that microorganisms dominate the diversity on Earth and the protists, a paraphyletic assemblage of single-celled organisms, represent a significant part of this diversity (Adl et al., 2019; Burki et al., 2020). Protists can be found in a variety of habitats, often representing the largest portion of eukaryotic richness (de Vargas et al., 2015; Mahé et al., 2017; Singer et al., 2019; Obiol et al., 2020). Although they are very common, present in virtually all environments, molecular surveys of biodiversity has revealed that most of the taxonomic diversity of protists remains undescribed (Bass & Boenigk, 2011; Pawlowski et al., 2012; del Campo et al., 2014). This is especially evident in less explored regions such as the Neotropics (Dunthorn et al., 2012; Lentendu et al., 2019; Fernandes et al., 2021; Ritter et al., 2021; Câmara et al., 2022). This gap in knowledge about the taxonomic and functional diversity of protists is an obstacle to a clearer view of how ecosystems operate (Sherr et al., 2007; López-García & Moreira, 2008).

Protists are key components in the ecosystems they inhabit. They can be found as free-living forms, but many species are symbionts (*i.e*., parasites, parasitoids, mutualists and commensals) on a wide range of hosts, including other protists, plants and metazoans, directly affecting the ecological aspects and controlling populations of their hosts (Chambouvet et al., 2008; Nowack & Melkonian, 2010; Edgcomb, 2016). These parasitic and mutualistic symbionts can dominate the diversity and abundance in several environments (Guillou et al., 2008; Geisen et al., 2015; Mahé et al., 2017). In addition to being a source of food for many organisms, protists act at the base of food chains as primary producers, as consumers of bacteria or other protists, and as decomposers (Wetzel, 2001; Corliss, 2002). There are also protists capable of performing both photosynthesis and phagotrophy, according to their life cycle stage or environmental conditions, in a type of nutrition called mixotrophy (Jones, 2001; Mitra et al., 2014). Despite the importance of understanding the functional profile of protist communities in different environments (Singer et al., 2021), this type of investigation has rarely been done in Brazilian ecosystems (de Araujo et al., 2018).

The constant variation of the physico-chemical conditions and an overlap with the microbial communities from the adjacent soil could favor the development of a highly diverse and dynamic protistan assemblage in freshwater systems (Debroas et al., 2017; Boenigk et al., 2018). However, molecular studies have shown that transitional environments, such as brackish coastal lagoons and estuaries, also have high protist diversity (Schubert et al., 2011; Telesh et al., 2011; Telesh, Schubert & Skarlato, 2013; Grinienė et al., 2019), contrary to what was previously believed (Remane, 1934).

High-throughput sequencing of molecular markers from environmental samples, known as metabarcoding, are powerful tools to describe the diversity of protists (de Vargas et al., 2015) and have expanded our knowledge about the phylogenetic placement of these organisms and uncovered a high number of new lineages (Jamy et al., 2020; Rajter & Dunthorn, 2021; Czech et al., 2022). In under-explored regions with high potential for discovering new taxa, such as the Brazilian biomes (Fernandes et al., 2021; Câmara et al., 2022), this tool is even more promising.

The Atlantic Forest is one of the top two Brazilian ecosystems richest in plant and animal diversity and endemism (Mittermeier et al., 1999) and the world’s fourth leading biodiversity hotspot (Myers et al., 2000). At the same time, this is one of the global most depleted habitats, retaining only a small part of its primary vegetation (Mittermeier et al., 1999). A number of associated habitats such as mangroves, rivers, streams, creeks, lakes, and lagoons are included in this biome. More than 90% of the Atlantic Forest is within the Brazilian territory, therefore, its conservation is largely a Brazilian concern (Marques & Grelle, 2021). While the diversity of plants and vertebrates is relatively well documented, little is known about its microbial diversity (Pontes, 2015; Ritter et al., 2021). To the best of our knowledge, only two article have been published so far on the molecular diversity of protists in the Brazilian Atlantic Forest through DNA metabarcoding and both dealt exclusively with the diversity of the phylum Ciliophora (Simão et al., 2017; Fernandes et al., 2021). This is the first study to examine the taxonomic and trophic diversity of the major protist groups in water bodies located in the Atlantic Forest by DNA metabarcoding. We compared the α and β diversity among samples for the overall protists communities and assessed the relative abundance of phototrophic, consumers, and parasitic taxa in brackish coastal lagoons and freshwater systems, also contributing to a better understanding of the dynamics and adaptations of protists to different salinity levels.

## Materials and Methods

### Sampling

**Figure 1:**
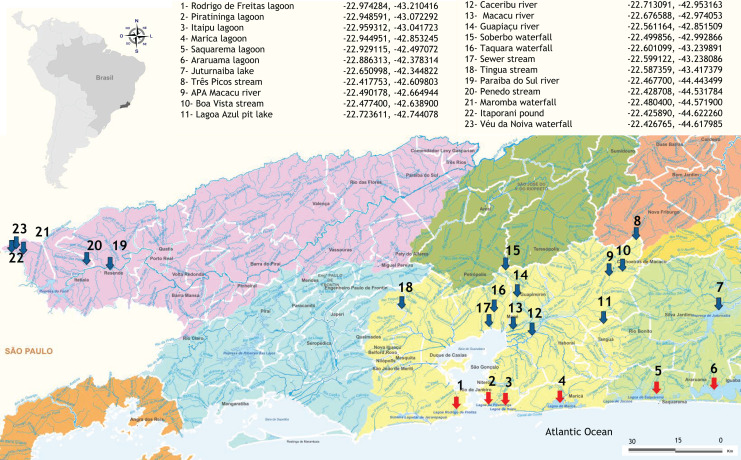
Distribution and geographical coordinates of the aquatic ecosystems investigated in fragments of Atlantic Forest, Rio de Janeiro State, Brazil. Red arrows indicate brackish coastal lagoons and blue arrows freshwater environments.

Samples of freshwater and brackish water were obtained from 23 sites located in fragments of the Atlantic Forest in Rio de Janeiro state, Brazil (Fig. 1), as detailed in Fernandes et al. (2021). Five aliquots of 200 mL of water and resuspended sediment were collected along the edges of each sampling site, making up a total volume of 1 L per sample. The samples were stored in sterile plastic containers and then taken to the laboratory for filtration and DNA extraction less than 24 h after sampling. The total volume was filtered with a peristaltic pump through 0.22 µm Polyethersulfone (PES) membranes (75 mm diameter) and the retained content (about 0.5 g) was immediately processed for DNA extraction, ensuring the integrity of the microbial community. Negative field controls (sterilized water collected using the same protocol and equipment) were also obtained and processed in the same way as field samples to monitor possible contamination.

### DNA extraction and Illumina library construction

Total DNA extraction was performed using the PowerSoil® DNA Isolation kit (MoBio Laboratories, Carlsbad, CA USA). DNA yields were measured using the Qubit® 2.0 Fluorometer (Thermo Scientific, Waltha, MA, USA). The universal primers 528F (5′-GCG GTA ATT CCA GCT CCA A-3′) and 706R (5′-AAT CCR AGA ATT TCA CCT CT-3′) (Elwood, Olsen & Sogin, 1985; Cheung et al., 2010) were used to amplify the V4 region of the eukaryotic 18S rRNA gene in PCR reactions with the Phusion® High-Fidelity PCR Master Mix (New England Biolabs, USA). The amplicons were sequenced with an Illumina HiSeq 2500 sequencer (Illumina Inc., San Diego, CA, USA), and 2 × 250 bp reads were generated. Raw sequences are available through the project number PRJEB37554 on the European Nucleotide Archive (ENA).

### Bioinformatics analyses

Sequencing reads from all samples were first merged with Flash v1.2.11 (Magoč & Salzberg, 2011) and then processed with Quantitative Insights Into Microbial Ecology 2—QIIME2 2022.2 (Bolyen et al., 2019) for demultiplexing and remotion of adaptors, using the q2-demux and q2-cutadapt (Martin, 2011) plugins. The reads were filtered to a minimum Phred quality score of Q20, denoised, dereplicated, and chimerical sequences were eliminated using the q2-quality-filter (Bokulich et al., 2013) and the q2-dada2 plugins (Callahan et al., 2016), respectively. Reads shorter than 210 bp length were also discarded. The amplicon sequence variants were clustered into operational taxonomic units (OTUs) using the q2-vsearch plugin (Rognes et al., 2016) and the open-reference method (Rideout et al., 2014) against the SILVA reference database version 138 (Quast et al., 2012). Sequences with ≥97% similarity were assigned to the same OTU. A sklearn classifier pre-trained on SILVA 138, region 515F/806R, was used to the taxonomic annotation of OTUs (Bokulich et al., 2018) with the q2-feature-classifier plugin (Pedregosa et al., 2011). OTUs from putative multicellular organisms (*i.e*., assigned to Metazoa, Embryophyta and Fungi) were removed, as well as the ones represented by less than 10 sequences, for noise reduction (Behnke et al., 2011).

### Functional assignments of OTUs

The obtained taxonomy table was manually verified and OTUs were assigned to three major functional groups following Singer et al. (2021) as consumers (Ciliophora, Rhizaria, Obazoa non-Ichthyosporea, CRUMs, Amoebozoa, non-Ochrophyta, non-Peronosporomycetes Stramenopiles and Centrohelida), phototrophic (Archaeplastida, Ochrophyta, Prymnesiophyceae and Cryptophyceae) and parasitic (Apicomplexa, Ichtyosporea, Peronosporomycetes, Phytomyxea, Perkinsidae, Syndiniales and Rozellomycota). Since these groups may include organisms with different functional roles, we analyze each OTU classified and consider the least inclusive taxonomic level to assign function (Table S1). Some groups of Chrysophyceae have lost their photosynthetic ability secondarily (Dorrell et al., 2019). Therefore, we considered as consumers those OTUs assigned to *Oikomonas*, *Spumella*, *Apoikia*, *Poteriospumella* and *Paraphysomonas*, also following Singer et al. (2021). Other genera were considered phototrophic and the OTUs not classified at this level were tagged with unknown function.

### Diversity studies

We estimated the α diversity, *i.e*., the number of observed OTUs, the Shannon’s index H′ (Whittaker, 1972), and the Simpson’s index D (Simpson, 1949) for each sample with the R-package phyloseq (McMurdie & Holmes, 2013). OTU richness in freshwater and brackish samples was also estimated using species accumulation curves (functions specaccum, R-package vegan v. 2.6–2) (Oksanen et al., 2022). We assessed the similarity patterns among protist communities (β diversity) using principal coordinate analysis (PCoA) based on Bray-Curtis dissimilarities obtained from the composition and relative abundance of sequences. Significance of differences between groups was assessed using the Permanova test (adonis function R-package vegan with 1,000 permutations). We tested for differences between ecosystems for α and β diversity indices by pairwise tests for multiple comparisons of mean rank sums (Nemenyi test, *p* < 0.05; function NemenyiTest, R package DescTools). We also use this approach to test the differences between functional groups based on the relative abundance of OTUs.

## Results

### Protist community richness and heterogeneity in freshwater and brackish systems from Atlantic Forest

The sequencing generated a total of 1,742,075 reads. After all quality filtering steps, 253,637 reads with an average sequence length of 350 bp remained for downstream analysis. After clustering at 97% similarity, a total of 2,692 OTUs were retrieved. Subsequently, OTUs not assigned to the phylum taxonomic category, identified as ‘unclassified’, ‘uncultured’ and ‘incertae sedis’ (256 OTUs) were removed, as well as sequences from putative non-protist organisms, as OTUs assigned to Metazoa (383 OTUs), Fungi (408 OTUs) and Embryophyta (55 OTUs). In the end, a total of 1,590 OTU sequences assigned to protist groups were retained (Table S1) and used for the diversity analyses.

**Figure 2:**
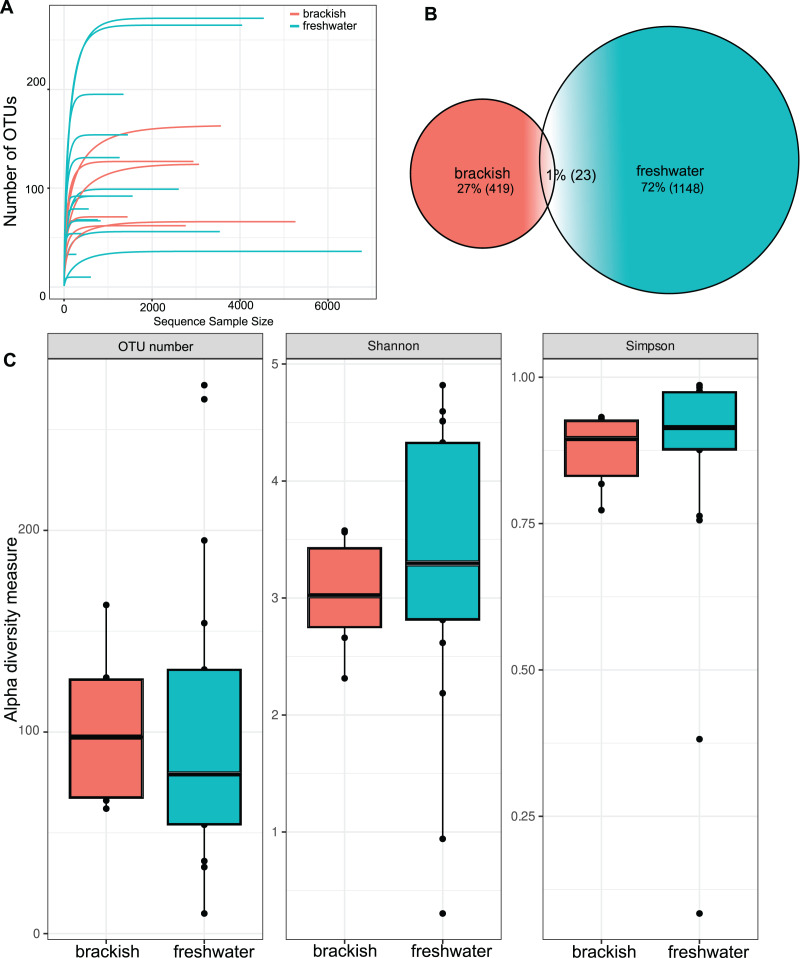
Richness and diversity of protist OTUs. (A) Species accumulation curves by sample. (B) Venn’s diagram of the total amount of OTUs in freshwater, brackish water and shared by both sample groups. (C) α diversity metrics. Number of unique OTUs; Shannon = Shannon’s index H; Simpson = Simpson’s index D. The average values of the α diversity indexes do not differ significantly between freshwater and brackish sampling groups (*p*-value > 0.05).

OTU richness tended to approach a saturation plateau, as shown by the species accumulation curves (Fig. 2A). Protist richness was significantly higher in freshwater (1,148 OTUs) than in brackish samples (419 OTUs). Only 23 OTUs were shared between these two sampling groups (Fig. 2B). However, when abundance data are considered, the means of the α diversity indices do not differ significantly between freshwater and brackish environments by t-test (Fig. 2C), suggesting that a more even distribution of sampling effort could equalize the OTU richness retrieved from these environments. The samples with the highest OTU richness were from Guapiaçu river (265 OTUs) and from Três Picos Park (272 OTUs). The sample with the highest α diversity value was from the Boa Vista stream (Table S2). All these highly diverse sites are located in the Serra dos Órgãos National Park.

**Figure 3:**
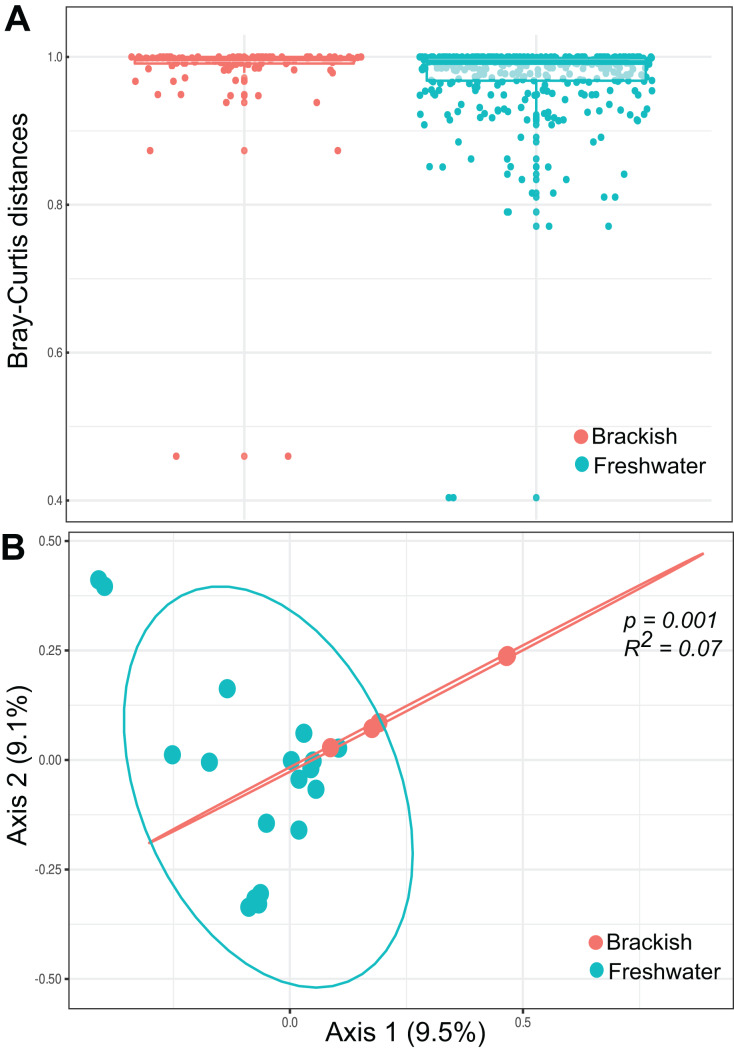
Beta diversity measures. (A) Bray-Curtis distances within each ecosystem based on protist OTU composition (presence-absence data) and relative abundances. (B) Ordination plot (principal coordinates analysis = PCoA) of protists communities based on Bray-Curtis dissimilarities. The protist OTU composition in freshwater and brackish samples differs significantly (*p*-value < 0.01).

Beta diversity was highest among freshwater samples (0.968 $\pm$ 0.06) and significantly lower among brackish water samples (0.911 $\pm$ 0.15) (Fig. 3A). Principal Coordinate Analysis revealed that protist communities from brackish and freshwater environments are distinctly structured (Fig. 3B). The adonis test showed that the richness and abundance of the protist OTUs are significantly different between freshwater and brackish samples (*p*-value < 0.01; Fig. 3B). This dissimilarity between the two environments with respect to the protist communities was also confirmed by the Nemenyi’s test for multiple comparisons (*p*-value = 0.0014).

### Taxonomic and functional diversity

The 1,590 OTUs were distributed among seven of the protist supergroups (*sensu* Burki et al., 2020). As expected, most of the sequences were assigned to the clade TSAR (1,292 OTUs), representing more than 80% of the total diversity. Archaeplastida (177 OTUs) followed with 11% of the total OTU diversity. The other groups were much less represented, such as Obazoa (44 OTUs), Amoebozoa (31 OTUs), Cryptista (23 OTUs), CruMs (13 OTUs), and Haptista (9 OTUs) (Table S1).

**Figure 4:**
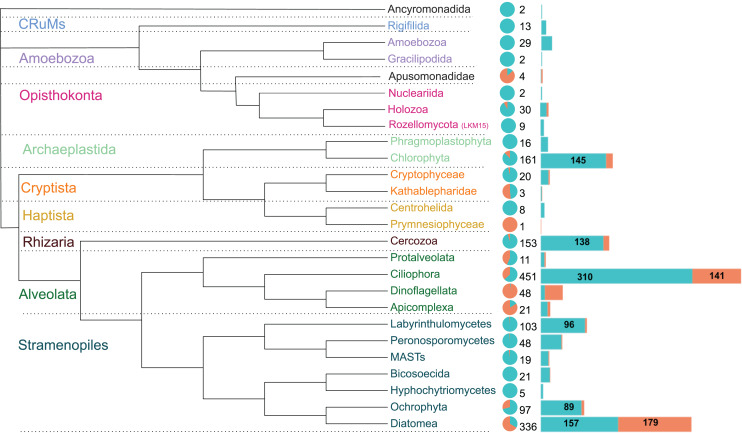
Schematic phylogenetic tree of the main protist lineages, their relative abundances and OTU richness in freshwater (cyan) and coastal (coral) aquatic ecosystems of the Atlantic Forest. The pie chart represents the relative abundance of reads. Numbers at the right of the pie chart are the total OTU richness of each taxon and the barplots represent the distribution of these OTUs in each ecosystem. Protist groups with highest OTUs richness are indicated numerically. Ciliophora dominates the diversity in freshwater systems while Diatomea is the richest and most abundant group in brackish waters.

These OTUs were assigned to 26 major protist phyla (Fig. 4). Ciliophora is the most represented (451 OTUs), followed by Diatomea (336 OTUs), Chlorophyta (161 OTUs) and Cercozoa (153 OTUs), together accounting for over two-thirds of the sequences. The relative abundance in brackish lagoons is dominated by Diatomea, Ciliophora and Dinoflagellata. Other major protist lineages are relatively more abundant or exclusive to freshwater (Fig. 4). Diatomea was the only group with higher OTU richness in brackish water (Fig. 4). The most represented at the genus level were the bacillariophycean diatoms *Navicula*, *Amphora*, *Pinnularia* and *Nitzschia* with more than 20 OTUs each (Table S1). A single OTU assigned to a marine haptophyte of the genus *Isochrysis* was detected exclusively in brackish samples (Table S1). From the total, only 418 OTUs were detected in relative abundances ≥1%. This represents 26.3% of the total data set (Table S3). Some of these OTUs showed relative abundances greater than 20% in the samples (Table 1). In particular, ciliates of the genera *Paramecium* and *Laurentiella* showed relative abundances of 96% and 80%, respectively, in some freshwater samples. Overall, ciliates are among the top five most abundant protists in the Brazilian Atlantic Forest (Fig. 4; Table S4).

**Table 1:** OTUs whose abundance exceeded 20% in the samples.

Protist main taxa	Identification at genus rank	Environment of highest abundance	Highest abundance (%)
Ciliophora	*Paramecium*	Freshwater	0.96
Ciliophora	*Laurentiella*	Freshwater	0.80
Ciliophora	*Frontonia*	Freshwater	0.65
Ciliophora	*Blepharisma*	Freshwater	0.53
Ciliophora	*Zoothamnium*	Brackish	0.48
Labyrinthulomycetes	*Labyrinthula*	Freshwater	0.46
Diatomea	*Synedra*	Freshwater	0.44
Dinoflagellata	*Blixaea*	Brackish	0.42
Phragmoplastophyta	*Spirogyra*	Freshwater	0.41
Ciliophora	*Heliophrya*	Freshwater	0.40
Diatomea	*Amphora*	Brackish	0.31
Diatomea	*Pleurosigma*	Brackish	0.27
Ciliophora	*Prorodon*	Freshwater	0.27
Diatomea	*Gyrosigma*	Brackish	0.25
Diatomea	*Stenopterobia*	Freshwater	0.22

**Figure 5:**
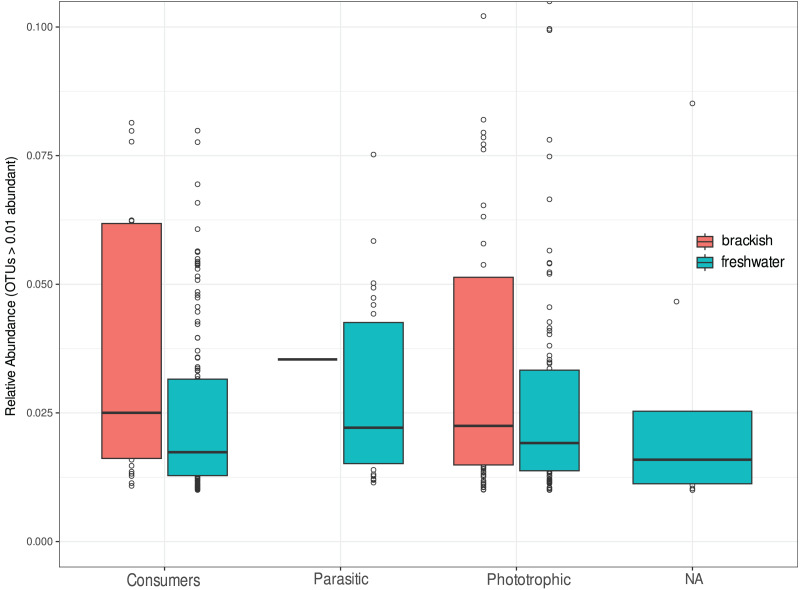
Relative abundance of OTUs assigned to consumers, parasitic or phototrophic protists in freshwater (cyan) and brackish (coral) aquatic systems of the Brazilian Atlantic Forest. OTUs representing groups of organisms that can functionally range from phototrophs to heterotrophs are indicated as “NA” (not assigned). Relative abundances do not differ statistically by functional group of protists in these ecosystems (Nemenyi test *p*-value > 0.05).

We investigate the functional diversity of protists in the two environments, expressed in relative abundance of consumers, phototrophics and parasites (Fig. 5). Of the total OTUs, 848 were attributed to consumers (51.5%), 602 to phototrophics (43.3%), 103 to parasites (5.25%), and 37 OTUs (5.5%) were assigned to groups of organisms that can functionally range from phototrophs to heterotrophs, so we cannot unambiguously assign their functional roles (Table S1). For statistical and graphical purposes, we considered only the 418 OTUs ≥1% abundant in the functional profile analyses (Table S3). Our results showed a remarkable functional homogeneity between the two ecosystems, with non-significant differences between them according to the Nemenyi test (*p*-value > 0.05). Consumers dominate the richness in freshwater, corresponding to more than 50% of the OTUs in this environment, while in brackish water there is a higher richness of phototrophic protists (Table 2), although the relative abundance of functional groups was statistically equivalent in both environments (Fig. 5).

**Table 2:** Distribution of the functional diversity of protists in the Brazilian Atlantic Forest.

Functional group	Ecosystem	Number of OTUs	Corresponding %
Consumer	Freshwater	172	41.1
Consumer	Brackish	26	6.2
Parasites	Freshwater	27	6.4
Parasites	Brackish	1	1.0
Phototrophics	Freshwater	111	26.5
Phototrophics	Brackish	68	16.2
NA	Freshwater	13	3.1
NA	Brackish	0	0

## Discussion

### Protist communities in coastal lagoons and freshwater systems of the Brazilian Atlantic Forest are equally diverse

The vast majority of biodiversity studies using HTS technology have been conducted in marine environments (*e.g*., Rychert et al., 2014; de Vargas et al., 2015; Massana et al., 2015; Gimmler et al., 2016). Relatively few metabarcoding surveys have been dedicated to investigating the diversity of inland waters, which are potentially much more diverse (*e.g*., Zinger, Gobet & Pommier, 2012; Balzano, Abs & Leterme, 2015; Fernandes et al., 2021). In understudied geographic regions, such as South America, these approaches are even rarer. We investigate for the first time the taxonomic and functional diversity of major protist lineages in freshwater and brackish systems located in fragments of the Brazilian Atlantic Forest. Specifically, the brackish systems studied are coastal lagoons, located in densely populated areas and considered one of the most impacted environments in the world (Esteves et al., 2008).

Our results showed that the diversity of protists in these coastal lagoons does not significantly differ from that in freshwater in terms of OTU richness and relative abundances, even though the number of samples analyzed from coastal lagoons is much smaller (six brackish *vs* 17 freshwater samples). This result is in contrast to Remane’s concept of a minimum number of species in transitional waters (Remane, 1934), which argues that taxonomic diversity is lowest at salinities between 5 and 8 psu (Kinne, 1971). However, this concept has been shown to be based on insufficient knowledge of the taxonomic composition of organisms (Telesh et al., 2011; Telesh, Schubert & Skarlato, 2011). Conversely, bacterial and protist diversity is usually higher in brackish waters (Telesh, Schubert & Skarlato, 2013; Santoferrara, Rubin & McManus, 2018) or comparable to other environments (*e.g*., Hu et al., 2016). Other diversity surveys in Brazilian coastal lagoons have reported high zooplankton diversity (Reid & Esteves, 1984; Branco, de Assis Esteves & Kozlowsky-Suzuki, 2000), with α diversity indexes comparable to that of Amazonian lakes (Carneiro, Bozelli & Esteves, 2003; Esteves et al., 2008). Here, we have observed the same pattern for protists.

Although our findings indicate that freshwater and brackish systems from Atlantic Forest are similar in terms of protist OTU richness and structure, including in relation to the functional profile of the organisms (details below), these two ecosystems differ significantly in terms of OTU taxonomic composition. Most OTUs were detected in either freshwater or brackish water, so the protist community composition differed significantly between the two environments (Fig. 3B). This was expected, as they are completely different ecosystems, and as previously reported for ciliates (Fernandes et al., 2021). However, a total of 23 OTUs were recorded in both ecosystems, including the bacillariophycean diatoms *Navicula*, *Amphora* and *Gomphonema*, and the ciliates *Paramecium* and *Laurentiella*, which can tolerate a wide range of salinity levels (Wilson, Cumming & Smol, 1996; Clavero et al., 2000; Smurov & Fokin, 2001).

Bray-Curtis distances were significantly greater among freshwater samples. This indicates greater heterogeneity within this sampling group in terms of OTU composition and abundance compared to brackish samples. This result was expected because the freshwater samples analyzed were taken from different water bodies, *i.e*., ponds, rivers, streams and waterfalls, which represent totally different environments, with different flow conditions, oxygen levels, *etc*. On the other hand, brackish coastal lagoons tend to be more similar to each other than to continental or marine waters, due to shared features such as strong physico-chemical gradients with adjacent ecosystems, variations in salinity and shallowness, among others (Pérez-Ruzafa et al., 2011), especially if they are geographically close and connected. Thus, these environments may share a basic set of species adapted to the same environmental conditions, or ecological guilds (Pérez-Ruzafa et al., 2011). However, due to the reduced number of brackish samples analyzed, this pattern should be considered with caution.

The freshwater samples located at Serra dos Órgãos National Park, a federal protected area (Rylands & Brandon, 2005), were the richest in protist OTUs and with the highest α diversity indexes in general. The potential of Brazilian protected areas for the discovery of new protistan taxa is underlined by the number of unclassified OTUs beyond class rank in these samples (Table S1). Indeed, several new protist taxa have recently been described from the same sampling ecosystems here investigated (*e.g*., Paiva et al., 2016; Campello-Nunes et al., 2015; Campello-Nunes et al., 2020; Campello-Nunes et al., 2022). This also emphasizes the importance of expanding sampling efforts in neotropical environments to enhance our comprehension of the global protist diversity.

### Functional groups are homogeneously represented in freshwater systems of the Brazilian Atlantic Forest

Ciliates have been the richest and relatively most abundant group in the studied freshwater ecosystems. These heterotrophic organisms have a wide range of life styles and have been successful in the colonization of diverse environments (Lynn, 2008). In fact, it is one of the most represented protist groups not only in the Atlantic Forest (Simão et al., 2017; Fernandes et al., 2021), but also in other Brazilian biomes (de Araujo et al., 2018; Lentendu et al., 2019). A previous study suggested that nearly one third of the ciliate OTUs share less than 97% sequence identity with reference sequences and may represent new ciliate taxa or nominal morphotypes that have already been described, but for which 18S rRNA gene sequences have not yet been deposited in reference databases (Fernandes et al., 2021). However, heterogeneity in rRNA copy numbers in ciliate macronuclei may overestimate their relative abundances (Gong et al., 2013; Geisen et al., 2015). The second most represented group overall and the only group with higher OTU richness and relative abundance in brackish water was Diatomea, mostly the photosynthetic Bacillariophyceae, also following previous surveys in estuaries and coastal lagoons (Roselli et al., 2013; Carstensen, Klais & Cloern, 2015; Leruste et al., 2019; Stefanidou et al., 2020). This success can be attributed to the ability of these organisms to adapt to the severe environmental fluctuations inherent to transitional environments (Snoeijs & Weckström, 2010).

Regarding the functional profile of protist communities, we detected a remarkable functional homogeneity between freshwater and brackish ecosystems, with non-significant differences between them in terms of relative abundances (Fig. 5). This means that there is no dominance of a specific functional group, with the proportions of consumers, phototrophics and parasites roughly balanced in the investigated freshwater environments. The same applies for the investigated brackish systems, in which the proportions of heterotrophic and phototrophic protists are equivalent. However, only a single OTU classified as an apicomplexan parasite was detected (Table S4), revealing low richness and abundance of protist parasites in brackish environments of the Atlantic Forest. Apicomplexa is an extremely diverse group and usually occur in high abundances in a variety of environments, including soils, most commonly infecting metazoans (Geisen et al., 2015; Mahé et al., 2017).

Heterotrophs protists contributed more to freshwater richness than phototrophs, contrary to previous studies (*e.g*., Singer et al., 2021; Garner et al., 2022). In marine waters there is also a predominance of consumers, as detected by the TARA Oceans expedition (de Vargas et al., 2015). In fact, heterotrophic protists act as primary consumers, transferring significant amounts of bacterial production to higher trophic levels, contributing to nutrient cycling in aquatic food webs (Azam et al., 1983), therefore are essential components of planktonic communities in aquatic systems in general (Nagata, 1986; Jürgens & Massana, 2008). However, we detected a higher richness of phototrophic protists in brackish systems compared to other trophic groups, suggesting a protagonist of microbial photosynthesis in this ecosystem. The functional roles of protists have been extensively studied in marine waters (*e.g*., Caron et al., 2012, 2017), and comparatively less investigated in continental environments, such as soils and freshwater (Geisen et al., 2018; Singer et al., 2021). Investigating the taxonomic and functional diversity of protists is essential to better understand the evolution, geographic distribution patterns, and ecological roles of these organisms in the Neotropics (Ritter et al., 2021), besides being the starting point for the development of public policies for sustainability and environmental protection. Overall, our study provides valuable information on the taxonomic and trophic profile of the protist communities from the freshwater and coastal brackish systems of the Brazilian Atlantic Forest.

## Supplemental Information

10.7717/peerj.15762/supp-1 Supplemental Information 1 Protist OTU Ids, their taxonomic and functional assignments.

10.7717/peerj.15762/supp-2 Supplemental Information 2 Alpha-diversity metrics by sample. Observed = number of unique OTUs; Shannon = Shannon’s index H; Simpson = Simpson’s index D.

10.7717/peerj.15762/supp-3 Supplemental Information 3 The OTUs that occur at above 1% of relative abundance in the investigated environments and their functional roles.

10.7717/peerj.15762/supp-4 Supplemental Information 4 Richness, relative and absolute abundances of the main protist lineages in freshwater and brackish systems of the Brazilian Atlantic Forest.

## Additional Information and Declarations

### Data Availability

The following information was supplied regarding data availability: The data is available at European Nucleotide Archive (ENA): PRJEB37554.

## References

[ref-1] Adl SM, Bass D, Lane CE, Lukeš J, Schoch CL, Smirnov A, Agatha S, Berney C, Brown MW, Burki F, Cárdenas P, Čepička I, Chistyakova L, Campo J, Dunthorn M, Edvardsen B, Eglit Y, Guillou L, Hampl V, Heiss AA, Hoppenrath M, James TY, Karnkowska A, Karpov S, Kim E, Kolisko M, Kudryavtsev A, Lahr DJG, Lara E, Le Gall L, Lynn DH, Mann DG, Massana R, Mitchell EAD, Morrow C, Park JS, Pawlowski JW, Powell MJ, Richter DJ, Rueckert S, Shadwick L, Shimano S, Spiegel FW, Torruella G, Youssef N, Zlatogursky V, Zhang Q (2019). Revisions to the classification, nomenclature, and diversity of eukaryotes. Journal of Eukaryotic Microbiology.

[ref-3] Azam F, Fenchel T, Field JG, Gray JS, Meyer-Reil LA, Thingstad F (1983). The ecological role of water-column microbes in the sea. Marine Ecology Progress Series. Oldendorf.

[ref-4] Balzano S, Abs E, Leterme SC (2015). Protist diversity along a salinity gradient in a coastal lagoon. Aquatic Microbial Ecology.

[ref-5] Bass D, Boenigk J (2011). Everything is everywhere: a twenty-first century de-/reconstruction with respect to protists. Biogeography of Microscopic Organisms.

[ref-6] Behnke A, Engel M, Christen R, Nebel M, Klein RR, Stoeck T (2011). Depicting more accurate pictures of protistan community complexity using pyrosequencing of hypervariable SSU rRNA gene regions. Environmental Microbiology.

[ref-7] Boenigk J, Wodniok S, Bock C, Beisser D, Hempel C, Grossmann L, Lange A, Jensen M (2018). Geographic distance and mountain ranges structure freshwater protist communities on a European scalе. Metabarcoding and Metagenomics.

[ref-8] Bokulich NA, Kaehler BD, Rideout JR, Dillon M, Bolyen E, Knight R, Huttley GA, Caporaso JG (2018). Optimizing taxonomic classification of marker gene amplicon sequences. PeerJ Preprints.

[ref-9] Bokulich NA, Subramanian S, Faith JJ, Gevers D, Gordon JI, Knight R, Mills DA, Caporaso JG (2013). Quality-filtering vastly improves diversity estimates from Illumina amplicon sequencing. Nature Methods.

[ref-10] Bolyen E, Rideout JR, Dillon MR, Bokulich NA, Abnet CC, Al-Ghalith GA, Alexander H, Alm EJ, Arumugam M, Asnicar F, Bai Y, Bisanz JE, Bittinger K, Brejnrod A, Brislawn CJ, Brown CT, Callahan BJ, Caraballo-Rodríguez AM, Chase J, Cope EK, Da Silva R, Diener C, Dorrestein PC, Douglas GM, Durall DM, Duvallet C, Edwardson CF, Ernst M, Estaki M, Fouquier J, Gauglitz JM, Gibbons SM, Gibson DL, Gonzalez A, Gorlick K, Guo J, Hillmann B, Holmes S, Holste H, Huttenhower C, Huttley GA, Janssen S, Jarmusch AK, Jiang L, Kaehler BD, Kang KB, Keefe CR, Keim P, Kelley ST, Knights D, Koester I, Kosciolek T, Kreps J, Langille MGI, Lee J, Ley R, Liu Y-X, Loftfield E, Lozupone C, Maher M, Marotz C, Martin BD, McDonald D, McIver LJ, Melnik AV, Metcalf JL, Morgan SC, Morton JT, Naimey AT, Navas-Molina JA, Nothias LF, Orchanian SB, Pearson T, Peoples SL, Petras D, Preuss ML, Pruesse E, Rasmussen LB, Rivers A, Robeson MS, Rosenthal P, Segata N, Shaffer M, Shiffer A, Sinha R, Song SJ, Spear JR, Swafford AD, Thompson LR, Torres PJ, Trinh P, Tripathi A, Turnbaugh PJ, Ul-Hasan S, van der Hooft JJJ, Vargas F, Vázquez-Baeza Y, Vogtmann E, von Hippel M, Walters W, Wan Y, Wang M, Warren J, Weber KC, Williamson CHD, Willis AD, Xu ZZ, Zaneveld JR, Zhang Y, Zhu Q, Knight R, Caporaso JG (2019). Reproducible, interactive, scalable and extensible microbiome data science using QIIME 2. Nature Biotechnology.

[ref-11] Branco CWC, de Assis Esteves F, Kozlowsky-Suzuki B (2000). The zooplankton and other limnological features of a humic coastal lagoon (Lagoa Comprida, Mace, RJ) in Brazil. Hydrobiologia.

[ref-12] Burki F, Roger AJ, Brown MW, Simpson AGB (2020). The new tree of eukaryotes. Trends in Ecology & Evolution.

[ref-13] Callahan BJ, McMurdie PJ, Rosen MJ, Han AW, Johnson AJA, Holmes SP (2016). DADA2: high-resolution sample inference from Illumina amplicon data. Nature Methods.

[ref-26] Câmara PEAS, Bones FLV, Lopes FAC, Oliveira FS, Barreto CC, Henriques DK, Campos LP, Carvalho-Silva M, Convey P, Rosa LH (2022). DNA metabarcoding reveals cryptic diversity in forest soils on the isolated Brazilian Trindade Island, South Atlantic. Microbial Ecology.

[ref-14] Campello-Nunes PH, Fernandes N, Schlegel M, Silva-Neto ID (2015). Description and phylogenetic position of *Corlissina maricaensis* gen. nov., sp. nov. (Karyorelictea, Geleiidae), a novel interstitial ciliate from Brazil, with redefinition of the family Geleiidae. International Journal of Systematic and Evolutionary Microbiology.

[ref-15] Campello-Nunes PH, Fernandes NM, Szokoli F, Fokin SI, Serra V, Modeo L, Petroni G, Soares CAG, Paiva TDS, Silva-Neto IDD (2020). *Parablepharisma* (Ciliophora) is not a heterotrich: a phylogenetic and morphological study with the proposal of new taxa. Protist.

[ref-16] Campello-Nunes PH, Silva-Neto ID, Sales MHO, Soares CAG, Paiva TS, Fernandes NM (2022). Morphological and phylogenetic investigations shed light on evolutionary relationships of the enigmatic genus *Copemetopus* (Ciliophora, Alveolata), with the proposal of *Copemetopus verae* sp. nov. European Journal of Protistology.

[ref-17] Carneiro LS, Bozelli RL, Esteves FA (2003). Long-term changes in the density of the copepod community in an Amazonian lake impacted by bauxite tailings. Amazoniana.

[ref-18] Caron DA, Connell PE, Schaffner RA, Schnetzer A, Fuhrman JA, Countway PD, Kim DY (2017). Planktonic food web structure at a coastal time-series site: I. Partitioning of microbial abundances and carbon biomass. Deep Sea Research Part I: Oceanographic Research Papers.

[ref-19] Caron DA, Countway PD, Jones AC, Kim DY, Schnetzer A (2012). Marine protistan diversity. Annual Review of Marine Science.

[ref-20] Carstensen J, Klais R, Cloern JE (2015). Phytoplankton blooms in estuarine and coastal waters: seasonal patterns and key species. Estuarine, Coastal and Shelf Science.

[ref-21] Chambouvet A, Morin P, Marie D, Guillou L (2008). Control of toxic marine dinoflagellate blooms by serial parasitic killers. Science.

[ref-22] Cheung MK, Au CH, Chu KH, Kwan HS, Wong CK (2010). Composition and genetic diversity of picoeukaryotes in subtropical coastal waters as revealed by 454 pyrosequencing. The ISME Journal.

[ref-23] Clavero E, Hernández-Mariné M, Grimalt JO, Garcia-Pichel F (2000). Salinity tolerance of diatoms from thalassic hypersaline environments. Journal of Phycology.

[ref-24] Corliss JO (2002). Biodiversity and biocomplexity of the protists and an overview of their significant roles in maintenance of our biosphere. Acta Protozoologica.

[ref-25] Czech L, Stamatakis A, Dunthorn M, Barbera P (2022). Metagenomic analysis using phylogenetic placement—a review of the first decade. Computational Methods for Microbiome Analysis.

[ref-27] de Araujo ASF, Mendes LW, Lemos LN, Antunes JEL, Beserra JEA, de Lyra MDCCP, Figueiredo MDVB, Lopes Ângela CDA, Gomes RLF, Bezerra WM, Melo VMM, de Araujo FF, Geisen S (2018). Protist species richness and soil microbiome complexity increase towards climax vegetation in the Brazilian Cerrado. Communications Biology.

[ref-28] de Vargas C, Audic S, Henry N, Decelle J, Mahé F, Logares R, Lara E, Berney C, Le Bescot N, Probert I, Carmichael M, Poulain J, Romac S, Colin S, Aury J-M, Bittner L, Chaffron S, Dunthorn M, Engelen S, Flegontova O, Guidi L, Horák A, Jaillon O, Lima-Mendez G, Lukeš J, Malviya S, Morard R, Mulot M, Scalco E, Siano R, Vincent F, Zingone A, Dimier C, Picheral M, Searson S, Kandels-Lewis S, Oceans Coordinators T, Gacinas S, Bork P, Bowler C, Gorsky G, Grimsley N, Hingamp P, Iudicone D, Not F, Ogata H, Pesant S, Raes J, Esieracki M, Speich S (2015). Eukaryotic plankton diversity in the sunlit ocean. Science.

[ref-29] Debroas D, Domaizon I, Humbert JF, Jardillier L, Lepére C, Oudart A, Taib N (2017). Overview of freshwater microbial eukaryotes diversity: a first analysis of publicly available metabarcoding data. FEMS Microbiology Ecology.

[ref-30] del Campo JD, Sieracki ME, Molestina R, Keeling P, Massana R, Ruiz-Trillo I (2014). The others: our biased perspective of eukaryotic genomes. Trends in Ecology and Evolution.

[ref-31] Dorrell RG, Azuma T, Nomura M, Audren de Kerdrel G, Paoli L, Yang S, Bowler C, Ishii K-I, Miyashita H, Gile GH, Kamikawa R (2019). Principles of plastid reductive evolution illuminated by nonphotosynthetic chrysophytes. Proceedings of the National Academy of Sciences of the United States of America.

[ref-32] Dunthorn M, Klier J, Bunge J, Stoeck T (2012). Comparing the hyper-variable V4 and V9 regions of the small subunit rDNA for assessment of ciliate environmental diversity. Journal of Eukaryotic Microbiology.

[ref-34] Edgcomb VP (2016). Marine protist associations and environmental impacts across trophic levels in the twilight zone and below. Current Opinion in Microbiology.

[ref-35] Elwood HJ, Olsen GJ, Sogin ML (1985). The small-subunit ribosomal RNA gene sequences from the hypotrichous ciliates *Oxytricha nova* and *Stylonychia pustulata*. Molecular Biology and Evolution.

[ref-36] Esteves FA, Caliman A, Santangelo JM, Guariento RD, Farjalla VF, Bozelli RL (2008). Neotropical coastal lagoons: an appraisal of their biodiversity, functioning, threats and conservation management. Brazilian Journal of Biology.

[ref-37] Fernandes NM, Campello-Nunes PH, Paiva TS, Soares CAG, Silva-Neto ID (2021). Ciliate diversity from aquatic environments in the Brazilian Atlantic Forest as revealed by high-throughput DNA sequencing. Microbial Ecology.

[ref-38] Garner RE, Kraemer SA, Onana VE, Huot Y, Gregory-Eaves I, Walsh DA (2022). Protist diversity and metabolic strategy in freshwater lakes are shaped by trophic state and watershed land use on a continental scale. mSystems.

[ref-39] Geisen S, Laros I, Vizcaíno A, Bonkowski M, De Groot G (2015). Not all are free-living: high-throughput DNA metabarcoding reveals a diverse community of protists parasitizing soil metazoa. Molecular Ecology.

[ref-40] Geisen S, Mitchell EAD, Adl S, Bonkowski M, Dunthorn M, Ekelund F, Fernández LD, Jousset A, Krashevska V, Singer D, Spiegel FW, Walochnik J, Lara E (2018). Soil protists: a fertile frontier in soil biology research. FEMS Microbiology Reviews.

[ref-42] Gimmler A, Korn R, de Vargas C, Audic S, Stoeck T (2016). The Tara Oceans voyage reveals global diversity and distribution patterns of marine planktonic ciliates. Scientific Reports.

[ref-43] Gong J, Dong J, Liu X, Massana R (2013). Extremely high copy numbers and polymorphisms of the rDNA operon estimated from single cell analysis of oligotrich and peritrich ciliates. Protist.

[ref-44] Grinienė E, Lesutienė J, Gorokhova E, Zemlys P, Gasiūnaitė ZR (2019). Lack of ciliate community integrity in transitional waters: a case study from the Baltic Sea. Estuarine, Coastal and Shelf Science.

[ref-45] Guillou L, Viprey M, Chambouvet A, Welsh RM, Kirkham AR, Massana R, Scanlan DJ, Worden AZ (2008). Widespread occurrence and genetic diversity of marine parasitoids belonging to *Syndiniales* (Alveolata). Environmental Microbiology.

[ref-46] Hu YO, Karlson B, Charvet S, Andersson AF (2016). Diversity of pico-to mesoplankton along the 2000 km salinity gradient of the Baltic Sea. Frontiers in Microbiology.

[ref-47] Jamy M, Foster R, Barbera P, Czech L, Kozlov A, Stamatakis A, Bending G, Hilton S, Bass D, Burki F (2020). Long metabarcoding of the eukaryotic rDNA operon to phylogenetically and taxonomically resolve environmental diversity. Molecular Ecology Resources.

[ref-48] Jones RI (2001). Mixotrophy in planktonic protists: an overview. Freshwater Biology.

[ref-49] Jürgens K, Massana R (2008). Protistan grazing on marine bacterioplankton. Microbial Ecology of the Oceans.

[ref-50] Kinne O (1971). Marine ecology. Environmental Factors, Part 2.

[ref-51] Lentendu G, Buosi PRB, Cabral AF, Trevizan Segóvia B, Ramos Meira B, Lansac‐Tôha FM, Velho LFM, Ritter CD, Dunthorn M (2019). Protist biodiversity and biogeography in lakes from four Brazilian river-floodplain systems. Journal of Eukaryotic Microbiology.

[ref-52] Leruste A, Pasqualini V, Garrido M, Malet N, De Wit R, Bec B (2019). Physiological and behavioral responses of phytoplankton communities to nutrient availability in a disturbed Mediterranean coastal lagoon. Estuarine, Coastal and Shelf Science.

[ref-54] López-García P, Moreira D (2008). Tracking microbial biodiversity through molecular and genomic ecology. Research in Microbiology.

[ref-53] Lynn D (2008). The ciliated protozoa: characterization, classification, and guide to the literature.

[ref-55] Magoč T, Salzberg SL (2011). FLASH: fast length adjustment of short reads to improve genome assemblies. Bioinformatics.

[ref-56] Mahé F, de Vargas C, Bass D, Czech L, Stamatakis A, Lara E, Singer D, Mayor J, Bunge J, Sernaker S, Siemensmeyer T, Trautmann I, Romac S, Berney C, Kozlov A, Mitchell EAD, Seppey CVW, Egge E, Lentendu G, Wirth R, Trueba G, Dunthorn M (2017). Parasites dominate hyperdiverse soil protist communities in neotropical rainforests. Nature Ecology and Evolution.

[ref-57] Marques MC, Grelle CE (2021). The Atlantic forest. History, Biodiversity, Threats and Opportunities of the Mega-diverse Forest.

[ref-102] Martin M (2011). Cutadapt removes adapter sequences from high-throughput sequencing reads. EMBnet.journal.

[ref-58] Massana R, Gobet A, Audic S, Bass D, Bittner L, Boutte C, Chambouvet A, Christen R, Claverie J-M, Decelle J, Dolan JR, Dunthorn M, Edvardsen B, Forn I, Forster D, Guillou L, Jaillon O, Kooistra WHCF, Logares R, Mahé F, Not F, Ogata H, Pawlowski J, Pernice MC, Probert I, Romac S, Richards T, Santini S, Shalchian-Tabrizi K, Siano R, Simon N, Stoeck T, Vaulot D, Zingone A, de Vargas C (2015). Marine protist diversity in European coastal waters and sediments as revealed by high-throughput sequencing. Environmental Microbiology.

[ref-59] McMurdie PJ, Holmes S (2013). phyloseq: an R package for reproducible interactive analysis and graphics of microbiome census data. PLOS ONE.

[ref-60] Mitra A, Flynn KJ, Burkholder JM, Berge T, Calbet A, Raven JA, Granéli E, Glibert PM, Hansen PJ, Stoecker DK, Thingstad F, Tillmann U, Våge S, Wilken S, Zubkov MV (2014). The role of mixotrophic protists in the biological carbon pump. Biogeosciences.

[ref-61] Mittermeier RA, Myers N, Mittermeier CG, Robles Gil P (1999). Hotspots: earth’s biologically richest and most endangered terrestrial ecoregions. http://www.jstor.org/stable/1383593.

[ref-62] Myers N, Mittermeier RA, Mittermeier CG, Da Fonseca GA, Kent J (2000). Biodiversity hotspots for conservation priorities. Nature.

[ref-63] Nagata T (1986). Carbon and nitrogen content of natural planktonic bacteria. Applied and Environmental Microbiology.

[ref-64] Nowack EC, Melkonian M (2010). Endosymbiotic associations within protists. Philosophical Transactions of the Royal Society B: Biological Sciences.

[ref-65] Obiol A, Giner CR, Sánchez P, Duarte CM, Acinas SG, Massana R (2020). A metagenomic assessment of microbial eukaryotic diversity in the global ocean. Molecular Ecology Resources.

[ref-66] Oksanen J, Simpson GL, Guillaume Blanchet F, Kindt R, Legendre P, Minchin PR, O’Hara RB, Solymos P, Henry M, Stevens H, Szoecs E, Wagner H, Barbour M, Bedward M, Bolker B, Borcard D, Carvalho G, Chirico M, de Caceres M, Durand S, Evangelista HBA, FitzJohn R, Friendly M, Furneaux B, Hannigan G, Hill MO, Lahti L, McGlinn D, Ouellette M-H, Cunha ER, Smith T, Stier A, Ter Braak CJF, Weedon J (2022). Vegan: community ecology package, 2.6–2. https://cran.r-project.org/web/packages/vegan/vegan.pdf.

[ref-67] Paiva TDS, Shao C, Fernandes NM, Borges BDN, Da Silva-Neto ID (2016). Description and phylogeny of *Urostyla grandis wiackowskii* subsp. nov. (Ciliophora, Hypotricha) from an estuarine Mangrove in Brazil. Journal of Eukaryotic Microbiology.

[ref-68] Pawlowski J, Audic S, Adl S, Bass D, Belbahri L, Berney C, Bowser SS, Cepicka I, Decelle J, Dunthorn M, Fiore-Donno AM, Gile GH, Holzmann M, Jahn R, Jirků M, Keeling PJ, Kostka M, Kudryavtsev A, Lara E, Lukeš J, Mann DG, Mitchell EAD, Nitsche F, Romeralo M, Saunders GW, Simpson AGB, Smirnov AV, Spouge JL, Stern RF, Stoeck T, Zimmermann J, Schindel D, de Vargas C (2012). CBOL protist working group: barcoding eukaryotic richness beyond the animal, plant, and fungal kingdoms. PLOS Biology.

[ref-69] Pedregosa F, Varoquaux G, Gramfort A, Michel V, Thirion B, Grisel O, Blondel M, Prettenhofer P, Weiss R, Dubourg V, Vanderplas J, Passos A, Cournapeau D, Brucher M, Perrot M, Duchesnay É (2011). Scikit-learn: machine learning in python. Journal of Machine Learning Research.

[ref-72] Pérez-Ruzafa A, Marcos C, Pérez-Ruzafa IM, Pérez-Marcos M (2011). Coastal lagoons: “transitional ecosystems” between transitional and coastal waters. Journal of Coastal Conservation.

[ref-71] Pontes JAL (2015). Biodiversidade Carioca: segredos revelados.

[ref-73] Quast C, Pruesse E, Yilmaz P, Gerken J, Schweer T, Yarza P, Peplies J, Glöckner FO (2012). The SILVA ribosomal RNA gene database project: improved data processing and web-based tools. Nucleic Acids Research.

[ref-74] Rajter Ľ, Dunthorn M (2021). Ciliate SSU-rDNA reference alignments and trees for phylogenetic placements of metabarcoding data. Metabarcoding and Metagenomics.

[ref-75] Reid J, Esteves FA (1984). Considerações ecológicas e biogeográficas sobre a fauna de copépodos (Crustacea) planctônicos e bentônicos de 14 lagoas costeiras do Estado do Rio de Janeiro, Brasil.

[ref-76] Remane A (1934). Die Brackwasserfauna: mit besonderer Berücksichtigung der Ostsee. https://oceanrep.geomar.de/id/eprint/43967.

[ref-77] Rideout JR, He Y, Navas-Molina JA, Walters WA, Ursell LK, Gibbons SM, Chase J, McDonald D, Gonzalez A, Robbins-Pianka A, Clemente JC, Gilbert JA, Huse SM, Zhou H-W, Rob Knight JGC (2014). Subsampled open-reference clustering creates consistent, comprehensive OTU definitions and scales to billions of sequences. PeerJ.

[ref-78] Ritter CD, Machado AF, Ribeiro KF, Dunthorn M (2021). Metabarcoding advances for ecology and biogeography of neotropical protists: what do we know, where do we go?. Biota Neotropica.

[ref-79] Rognes T, Flouri T, Nichols B, Quince C, Mahé F (2016). VSEARCH: a versatile open source tool for metagenomics. PeerJ.

[ref-80] Roselli L, Stanca E, Ludovisi A, Durante G, Souza JSD, Dural M, Alp T, Bulent S, Gjoni V, Ghinis S, Basset A (2013). Multi-scale biodiverity patterns in phytoplankton from coastal lagoons: the Eastern Mediterranean. Transitional Waters Bulletin.

[ref-81] Rychert K, Nawacka B, Majchrowski R, Zapadka T (2014). Latitudinal pattern of abundance and composition of ciliate communities in the surface waters of the Atlantic Ocean. Oceanological and Hydrobiological Studies.

[ref-82] Rylands AB, Brandon K (2005). Unidades de conservação brasileiras. Megadiversidade.

[ref-83] Santoferrara LF, Rubin E, McManus GB (2018). Global and local DNA (meta) barcoding reveal new biogeography patterns in tintinnid ciliates. Journal of Plankton Research.

[ref-84] Schubert H, Feuerpfeil P, Marquardt R, Telesh I, Skarlato S (2011). Macroalgal diversity along the Baltic Sea salinity gradient challenges Remane’s species-minimum concept. Marine Pollution Bulletin.

[ref-85] Sherr BF, Sherr E, Caron DA, Vaulot D, Worden AZ (2007). Oceanic protists. Oceanography.

[ref-87] Simão TLL, Borges AG, Gano KA, Davis-Richardson AG, Brown CT, Fagen JR, Triplett EW, Dias R, Mondin CA, da Silva RM, Eizirik E, Utz LRP (2017). Characterization of ciliate diversity in bromeliad tank waters from the Brazilian Atlantic Forest. European Journal of Protistology.

[ref-86] Simpson EH (1949). Measurement of diversity. Nature.

[ref-88] Singer D, Metz S, Unrein F, Shimano S, Mazei Y, Mitchell EAD, Lara E (2019). Contrasted micro-eukaryotic diversity associated with sphagnum mosses in tropical, subtropical and temperate climatic zones. Microbial ecology.

[ref-89] Singer D, Seppey CVW, Lentendu G, Dunthorn M, Bass D, Belbahri L, Blandenier Q, Didier Debroas GAG, de Vargas C, Domaizon I, Duckert C, Izaguirre I, Koenig I, Gabriela Mataloni MRS, Mitchell EAD, Geisen S, Lara E (2021). Protist taxonomic and functional diversity in soil, freshwater and marine ecosystems. Environment International.

[ref-90] Smurov AO, Fokin SI (2001). Use of salinity tolerance data for investigation of phylogeny of *Paramecium* (Ciliophora, Peniculia). Protistology.

[ref-91] Snoeijs P, Weckström K, Smol JP, Stoermer EF (2010). Diatoms and environmental change in large brackish-water ecosystems. The Diatoms: Applications for the Environmental and Earth Sciences.

[ref-92] Stefanidou N, Katsiapi M, Tsianis D, Demertzioglou M, Michaloudi E, Moustaka-Gouni M (2020). Patterns in α and β phytoplankton diversity along a conductivity gradient in coastal mediterranean lagoons. Diversity.

[ref-94] Telesh IV, Schubert H, Skarlato SO (2011). Revisiting Remane’s concept: evidence for high plankton diversity and a protistan species maximum in the horohalinicum of the Baltic Sea. Marine Ecology Progress Series.

[ref-95] Telesh IV, Schubert H, Skarlato S (2013). Life in the salinity gradient: discovering mechanisms behind a new biodiversity pattern. Estuarine, Coastal and Shelf Science.

[ref-96] Telesh IV, Schubert H, Skarlato SO, Ptacnik R, Olli K, Lehtinen S, Tamminen T, Andersen T (2011). Protistan diversity does peak in the horohalinicum of the Baltic Sea: reply to Ptacnik, R., Olli, K., Lehtinen, S., Tamminen, T & Andersen, T. (2011)—does plankton diversity peak at intermediate salinities?. Marine Ecology Progress Series.

[ref-97] Wetzel RG (2001). Protists: key ecosystem regulators. BioScience.

[ref-98] Whittaker RH (1972). Evolution and measurement of species diversity. Taxon.

[ref-99] Wilson SE, Cumming BF, Smol JP (1996). Assessing the reliability of salinity inference models from diatom assemblages: an examination of a 219-lake data set from western North America. Canadian Journal of Fisheries and Aquatic Sciences.

[ref-101] Zinger L, Gobet A, Pommier T (2012). Two decades of describing the unseen majority of aquatic microbial diversity. Molecular Ecology.

